# Effects of concentration-dependent graphene on maize seedling development and soil nutrients

**DOI:** 10.1038/s41598-023-29725-3

**Published:** 2023-02-14

**Authors:** Shiya Wang, Ying Liu, Xinyi Wang, Hongtao Xiang, Deyong Kong, Na Wei, Wei Guo, Haiyan Sun

**Affiliations:** 1 College of Agriculture, Heilongjiang Bayi Agriculture University, Daqing, 163319 China; 2 Heilongjiang Provincial Key Laboratory of Modern Agricultural Cultivation and Germplasm Improvement, Daqing, 163319 China; 3 Key Laboratory of Low Carbon Green Agriculture in Northeast Plain, Ministry of Agriculture and Rural Affairs, Daqing, 163319 Heilongjiang China; 4 Suihua Branch, Heilongjiang Academy of Agricultural Machinery Sciences, Suihua, 152054 China

**Keywords:** Plant sciences, Environmental sciences

## Abstract

The long-term use of chemical fertilizers to maintain agricultural production has had various harmful effects on farmland and has greatly impacted agriculture's sustainable expansion. Graphene, a unique and effective nanomaterial, is used in plant-soil applications to improve plant nutrient uptake, reduce chemical fertilizer pollution by relieving inadequate soil nutrient conditions and enhance soil absorption of nutrient components. We investigated the effects of graphene amendment on nutrient content, maize growth, and soil physicochemical parameters. In each treatment, 5 graphene concentration gradients (0, 25, 50, 100, and 150 g kg^−1^) were applied in 2 different types (single-layer and few-layers, SL and FL). Soil aggregates, soil accessible nutrients, soil enzyme activity, plant nutrients, plant height, stem diameter, dry weight, and fresh weight were all measured throughout the maize growth to the V3 stage. Compared to the control (0 g kg^−1^), we found that graphene increased the percentage of large agglomerates (0.25–10 mm) in the soil and significantly increased the geometric mean diameter (GMD) and mean weight diameter (MWD) values of > 0.25 mm water-stable agglomerates as the increase of concentration. Soil available nutrient content (AN, AP, and AK) increased, peaking at 150 g kg^−1^. Graphene boosted nutrient absorption by maize plants, and aboveground total nitrogen (TN), total phosphorus (TP), and total potassium (TK) contents rose with the increasing application, which raised aboveground fresh weight, dry weight, plant height, and stalk thickness. The findings above confirmed our prediction that adding graphene to the soil may improve maize plant biomass by enhancing soil fertility and improving the soil environment. Given the higher manufacturing cost of single-layer graphene and the greater effect of few-layer graphene on soil and maize plants at the same concentration, single-layer graphene and few-layer graphene at a concentration of 50 g kg^−1^ were the optimal application rates.

## Introduction

Black soil is a valuable non-renewable resource. The Northeast Plain of China is one of the world's three largest phaeozem zones and the heart of China's maize belt, accounting for around 30% of the country's total maize productivity and named China's Golden-Maize-Belt^1,2^. The soil types in this area mainly include mollisol, chernozem soil, dark brown soil, brown soil, albic soil, and meadow soil. Black soil possesses black or dark black humus, which is favorable for plant development due to its special characteristics like high fertility, strong agglomeration structure, water, fertilizer, air, heat coordination, etc.^3^. However, over-cultivation and climate change resulted in a series of degradation processes, affecting long-term agricultural development in China^4^. As a type of phaeozem, albic soil is mainly distributed in the northeastern part of Heilongjiang and Jilin provinces, with a total area of about 4.18 × 10^5^ ha^5^, which is crucial in the production of maize. Considering the physical property (tight compacted physical structure, hard heavy soil texture, and thin black soil layer), albic soil was regarded as one low-yielding soil type^6^. Despite the fact that chemical fertilizer application is currently one of the most important techniques for increasing yields in the region, it can cause hazards such as soil nutrient structure disorders, deterioration of physical properties, and plant growth due to the disadvantages of low utilization of traditional fertilizers and serious environmental pollution^7–9^. Therefore, speeding up the development of new materials and technologies with higher performance and applying them to maize production is a key problem that must be solved immediately in contemporary agriculture.

Nanotechnology has steadily been used for improving agricultural productivity in recent years. Nanotechnology ushers in a new solution in fertilization and environmental remediation due to its effectiveness in nutrition support and cost^10–13^. It was characterized by an enormous number of holes and wide surface area, as well as its lightweight, high electrical conductivity, and high strength^14,15^. The addition of graphene nanoparticles to fertilizers can increase soil clay content, improve soil texture, and improve the soil's capacity to absorb and store nutrients, thus reducing nutrient loss by above-ground volatilization, surface runoff, and deep seepage^16,17^. Simultaneously, it can enhance the electrochemical characteristics of the soil and stimulate nutrient absorption by the root system, boosting fertilizer use rates, lowering agricultural surface pollution, and contributing to fertilizer conservation and efficiency. In plant research, graphene and its derivatives can be applied as excellent water transporters in the soil to speed water intake by seeds, boost seed germination, and stimulate plant growth and development^18–20^. Inducing root growth WRKY genes, which enhances plant root biomass accumulation and supports the growth and development of above-ground organs, graphene may also boost plant root hair and lateral root development and promote root elongation^21–25^. Low concentrations of sulfonated graphene scavenged the formation of reactive oxygen species (ROS) in maize plants, improving root morphology and plant health in experiments^26^. Chakravarty et al.^27^ reported that the addition of graphene quantum dots to the soil, the purity, aggregation, and presence of various chemical functions on their outer walls will significantly affect their ability to interact with plant cells and influence seed germination, plant growth rate and protein production that is essential for plant development. On the contrary, several related studies have also summarized the potential toxic mechanisms, such as causing increased ROS, inhibiting antioxidant enzyme activity, causing metabolic disorders in the antioxidant system, and reducing chlorophyll biosynthesis^28^. Liu et al.^22^ reported that high concentrations of graphene (100–200 mg L^−1^) inhibit the growth of rice plants and reduce biomass. However, the mechanism of the effect of nanomaterials on plants is complex because many factors need to be considered, such as the characteristics of nanomaterials (type, concentration and surface characteristics) and plants (type and age)^29^.

In conclusion, there are disagreements concerning the impacts of graphene on plant growth and development. Further, research on the effects of graphene on soil physicochemical qualities is also limited. Therefore, the main objective of this study was to investigate the effect of graphene on soil physicochemical properties and plant growth. Here, we measured soil bulk structure, soil available nutrient content, soil enzyme activities, nutrient accumulation in the upper part of the maize ground, and carbon and metabolic indicators. This study helps elucidate graphene’s potential effects on soil physical properties and plant growth.

## Materials and methods

### General experimental conditions

Pot experiments were done in greenhouses at Heilongjiang Bayi Agricultural University's College of Agriculture in 2021 and 2022. (BYAU). The greenhouse's diurnal air temperature ranged from 23 to 28 °C/20 to 25 °C, and the intensity of light was 18,000 lx. The mollicplanosol was obtained from the experimental base of the Agricultural Research Institute of Heilongjiang Bayi Agricultural University, Mishan, Heilongjiang Province (131.8754° N, 46.5936° E). The soil’s chemical properties include available nitrogen (AN) content of 88.75 mg kg^−1^, available phosphorus (AP) content of 48.69 mg kg^−1^, and available potassium (AK) content of 94.01 mg kg^−1^, Total Organic Carbon (TOC) content of 15.6 g kg^−1^, soil organic matter (OM) content of 21.33 g kg^−1^, pH 5.67 (water–soil ratio 2.5:1).

Single-layer graphene and few-layer graphene were purchased from Shuncheng Chemical Products Trading Company, Zhengzhou, as black fluffy powder. The diameters of the single-layer graphene flakes were 0.5–10 μm and the diameters of the few-layer graphene flakes were 10–20 μm, and the average thicknesses were 1–3 nm.

The experimental fertilizers Urea (N 46%), diammonium phosphate (N 18%, P_2_O_5_ 46%), and potassium sulfate (K_2_O 50%) were purchased from the Yuntianhua group, Heilongjiang.

### Plant materials and treatments

Seeds were surface-sterilized with 10% sodium hypochlorite for 3 min and thoroughly washed with distilled water 3 times, and immersed in distilled water for 10 h. Then two seeds were planted in each plastic pot (upper diameter of 15 cm, height of 13 cm, water outlet at the bottom) filled with about 1.5 kg of soil and different types and concentrations of graphene, then mix well with fertilizer (the concentrations of urea, diammonium phosphate, and potassium sulfate were 375, 225, and 150 kg hm^2^, respectively). The experimental design is shown in Table 1. Experimental applications included: (1) Control without graphene addition (0 g kg^−1^); (2) Add 25 g kg^−1^ single layer graphene or few-layer graphene treatment, notated as SL1 and FL1; (3) Add 50 g kg^−1^ single layer graphene or few-layer graphene treatment, notated as SL2 and FL2; (4) Add 100 g kg^−1^ single layer graphene or few-layer graphene treatment, notated as SL3 and FL3; (5) Add 150 g kg^−1^ single layer graphene or few-layer graphene treatment, notated as SL4 and FL4; The test was arranged in a completely random design with four replicates for each treatment. When seeding, add 1.5 L of distilled water per pot and replace 0.5 L of distilled water every 3 days following seedling emergence. Plant and soil sampling was done after 30 days of plant emergence.

**Table 1:** Experimental treatment.

Graphene	Treatment	Concentration (g kg^−1^)
Single-layer graphene	CK	0
	SL1	25
	SL2	50
	SL3	100
	SL4	150
Few-layer graphene	CK	0
	FL1	25
	FL2	50
	FL3	100
	FL4	150

### Soil aggregate analysis

Soil samples were taken with a soil auger from the surface to the bottom layer of about 10 cm, dried, and weighed to 150 g. The mixed aggregate sample was sieved over the same set of sieves (5.00, 2.00, 1.00, 0.50, and 0.25 mm) while immersed in water (wet-sieving). The set of sieves was shaken up and down in the water for 30 min at the frequency of 30 beats min^−1^ (DM200-II aggregate analyzer, Shanghai, 2021). The water-stable agglomerates on each layer's sieve surface are then washed individually into the aluminum box, precipitated to remove the upper layer of water, dried, and weighed. Equations (1) to (4) for the indicators of aggregate structural stability such as detailed calculation of mean weight diameter (MWD), geometric mean diameter (GMD), water stable aggregates (WSA), Unstable Aggregate Index (*E*_*LT*_), and Fractal Dimension (*D*)^30,31^:

1 $$MWD=\frac{{\sum }_{i=1}^{n}{\overline{m} }_{i}{d}_{i}}{\sum_{i=1}^{n}{m}_{i}}$$

2 $$GMD=Exp\frac{\sum_{i=1}^{n}({m}_{i}ln\overline{{d }_{i})}}{\sum_{i=1}^{n}{m}_{i}}$$

3 $${E}_{LT}=\frac{{M}_{T}-{R}_{0.25}}{{M}_{T}}\times 100\%$$

4 $$\frac{M(r<{\overline{m} }_{i})}{{M}_{T}}={\left(\frac{{\overline{m} }_{i}}{{x}_{max}}\right)}^{3-D}$$

5 $$WSA=\frac{{R}_{0.25}}{{M}_{T}}$$

where *m*_*i*_ is the mass of aggregate fraction i (g), *d*_*i*_ is the mean diameter of the aggregate fraction i (mm), *n* denotes the number of aggregate size fractions, *M*(*r* ≤ *m*_*i*_) is the weight of aggregates with a fraction diameter less than or equal to *m*_*i*_, *M*_*T*_ is the gross weight of aggregates, and *R*_*0.25*_ is macroaggregates with diameters of > 0.25 mm.

### AN, AP, and AK of soil

Four replicated, undisturbed soil samples were taken from each pot at depths of 0–10 cm by soil sampler after removing all visible plant residues. All soil samples were stored at 4 °C and transported to the laboratory in aluminum containers within two days.

The alkali-dissolution diffusion method was used to evaluate soil AN, and the neutral NH_4_-Ac leaching-flame photometric colorimetric method was used to assess soil AK^32^ method. Soil AP was measured by treatment with 0.5 mol L^−1^ NaHCO_3_ followed by molybdenum blue colorimetry^33^.

### The activities of invertase, urease, and acid phosphatase in soil

Soil urease and sucrase activities were determined by the indophenol blue colorimetric method and the 3,5-dinitrosalicylic acid colorimetric method, respectively^34^.

### TN, TP, and TK content of the plant

1 g of the plant test sample in a digestion tube, add 10 mL of nitric acid, 1 mL of perchloric acid, and 2 mL of sulfuric acid, and digest on an adjustable electric heater. After cooling, transfer to a 100 mL volumetric flask and set the volume to the scale.

TN, TP, and TK contents of the plant were determined by Kjeldahl nitrogen determination, vanadium-molybdenum yellow colorimetric method, and flame photometer method, respectively^35^.

### Determination of plant height, stem diameter, fresh weight, and dry weight

Plant height and stalk thickness were measured using a straightedge and vernier caliper. Meanwhile, the above-ground fresh weight of maize seedlings was weighed, followed by killing at 105 °C for 60 min, drying at 80 °C to a constant weight, and weighing the dry weight, accurate to 0.001 g.

### Plant nutrient accumulation

Nutrient accumulation per plant was determined using plant dry weight and nutrient content.

$$\begin{gathered} {\text{N accumulation }}\left( {{\text{g}}\;{\text{plant}}^{{ - {1}}} } \right) = {\text{plant dry weight}} \times {\text{TN content}} \hfill \\ {\text{P accumulation }}\left( {{\text{g}}\;{\text{plant}}^{{ - {1}}} } \right) = {\text{plant dry weight}} \times {\text{TP content}} \hfill \\ {\text{K accumulation }}\left( {{\text{g}}\;{\text{plant}}^{{ - {1}}} } \right) = {\text{plant dry weight}} \times {\text{TK content}} \hfill \\ \end{gathered}$$

### Data analysis

Data statistics and analysis were performed using Microsoft Excel 2013 (Microsoft, Inc., red-mond WA, USA,) and SPSS 25.0 (SPSS software, 25.0, SPSS Institute Ltd, Chicago, USA). Differences among treatments were evaluated by one-way analysis of variance (ANOVA), Duncan’s test (*p* < 0.05) was used to evaluate the difference within treatments, and the significant differences among different materials were determined.

### Ethical approval

We state that our experimental research Queryon maize complies with the relevant institutional, national, and international guidelines and legislation.

## Result

### Soil aggregate-size distribution

**Figure 1:**
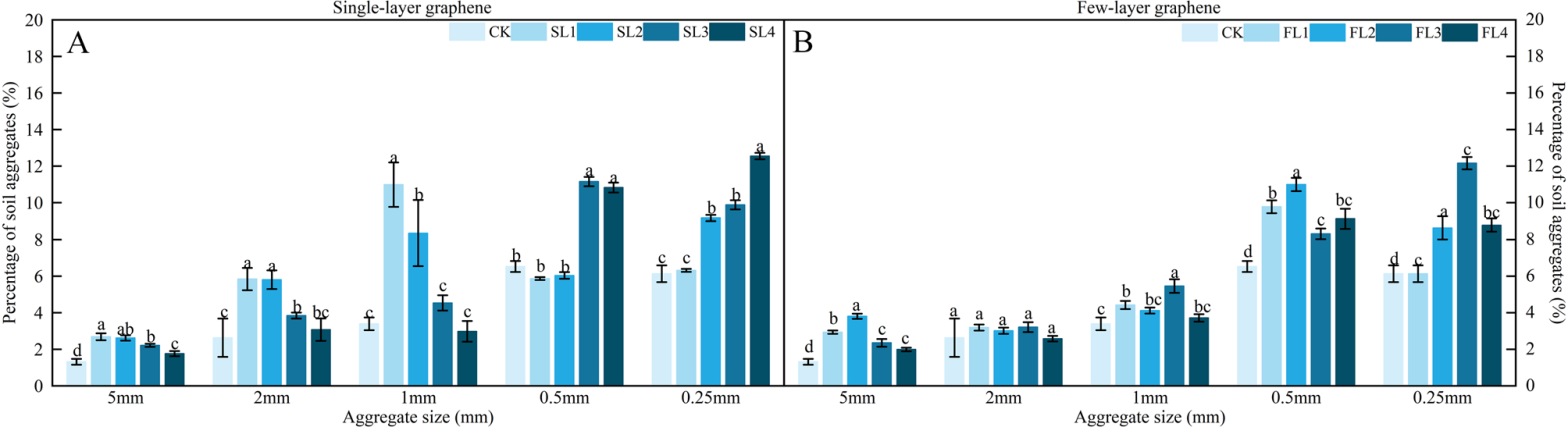
Effect of different graphene concentration and type on soil aggregate size distribution. CK. 0 g kg^−1^ single-layer graphene and 0 g kg^−1^ few-layer graphene; SL1 and FL1. 25 g kg^−1^ single-layer graphene and 25 g kg^−1^ few-layer graphene; SL2 and FL2. 50 g kg^−1^ single-layer graphene and 50 g kg^−1^ few-layer graphene; SL3 and FL3. 100 g kg^−1^ single-layer graphene and 100 g kg^−1^ few-layer graphene; SL4 and FL4. 150 g kg^−1^ single-layer graphene and 150 g kg^−1^ few-layer graphene; error bars indicate ± standard deviation. Different letters above error bars indicate significant difference between treatment (p < 0.05, LSD test).

The effects of different graphene on aggregate size distribution are shown in Fig. 1. Compared to CK, SL1, SL2, SL3, and SL4 effectively increased the content of > 1 mm agglomerates, with all treatments except SL4 reaching significant levels of difference, and the rise was more pronounced in SL1 and SL2 treatments (Fig. 1A). Agglomerate size reached the same level as CK for treatments SL1 and SL2 at < 0.5 mm and > 0.25 mm, while treatments SL3 and SL4 showed a significant increase compared to CK. At < 0.25 mm agglomerate size, the SL2, SL3, and SL4 treatments were significantly different from CK, and the SL4 treatment was significantly higher than the SL3 and SL2 treatments, while the SL1 treatment was not significantly different from CK.

In the few-layer graphene treatments (Fig. 1B), the FL1, FL2, FL3, and FL4 treatments increased the content of agglomerates by > 5 mm compared to CK, reaching significant difference levels between the sites. The treatments did not reach the level of significant difference compared to CK although there was an increase at the agglomerate sizes of 2 mm. At agglomerate sizes < 1 mm, there was a significant increase in FL1 and FL2 treatments compared to CK, and no significant difference in FL2 and FL4 compared to CK. At agglomerate sizes < 0.5 mm, all treatments were higher than the CK treatment by a significant difference level.

Table 2 shows that the graphene treatment considerably raised the WSA larger than 0.25 mm as well as the MWD and GMD values when compared to CK. For SL1, SL2, SL3, and SL4 treatments, the GMD rose by 51.32%, 46.05%, 36.84%, and 19.74%, respectively, while the MWD increased by 123.34%, 106.61%, 67.32%, and 34.24%. Compared to CK, FL1, FL2, FL3, and FL4 treatments substantially raised GMD by 34.87%, 38.16%, 26.32%, and 16.45%, respectively, and MWD by 74.32%, 96.50%, 47.86%, and 28.02%. It's also worth noting that the greater the MWD and GMD values, the lower the *E*_*LT*_ and *D*. When comparing with CK, the *E*_*LT*_ and *D* were lowered by 3.34–7.33% and 4.39–9.96% for the few-layer graphene treatment and 2.73–6.86% and 2.73–5.13% for the single-layer graphene treatment, respectively. It demonstrates that graphene can improve the stability of soil aggregates. The impact of single-layer graphene was superior to that of few-layer graphene.

**Table 2:** Effect of different graphene concentration and type on soil water-stable aggregate stability indexes.

Graphene	Treatment	WSA (%)	GMD (mm)	MWD (mm)	*E*_*LT*_/%	*D*
Single-layer graphene	SL1	0.139 ± 0.003d	0.152 ± 0.007d	0.257 ± 0.027d	93.864 ± 0.917a	3.689 ± 0.057a
	SL2	0.254 ± 0.010a	0.230 ± 0.008a	0.574 ± 0.057a	93.680 ± 0.163a	3.355 ± 0.030d
	SL3	0.228 ± 0.007b	0.222 ± 0.004a	0.531 ± 0.022a	90.827 ± 0.328b	3.381 ± 0.023d
	SL4	0.218 ± 0.002b	0.208 ± 0.003b	0.430 ± 0.016b	90.120 ± 0.502b	3.469 ± 0.010c
	CK	0.186 ± 0.007c	0.182 ± 0.013c	0.345 ± 0.089c	87.450 ± 0.349c	3.534 ± 0.045b
Few-layer graphene	FL1	0.139 ± 0.003d	0.152 ± 0.007d	0.257 ± 0.027e	93.864 ± 0.917a	3.689 ± 0.057a
	FL2	0.203 ± 0.008ab	0.205 ± 0.005a	0.448 ± 0.016b	91.366 ± 1.254b	3.528 ± 0.025bc
	FL3	0.219 ± 0.011a	0.210 ± 0.006a	0.505 ± 0.024a	87.840 ± 0.677d	3.509 ± 0.027c
	FL4	0.193 ± 0.017bc	0.192 ± 0.003b	0.380 ± 0.021c	91.213 ± 0.706b	3.532 ± 0.045bc
	SL1	0.174 ± 0.018c	0.177 ± 0.008c	0.329 ± 0.020d	89.707 ± 0.632c	3.591 ± 0.049b

*WSA* water stable aggregates, *GMD* geometric mean diameter, *MWD* mean weight diameter, *E*_*LT*_ unstable aggregate index, *D* fractal dimension, CK. 0 g kg^−1^ single-layer graphene and 0 g kg^−1^ few-layer graphene; SL1 and FL1. 25 g kg^−1^ single-layer graphene and 25 g kg^−1^ few-layer graphene; SL2 and FL2. 50 g kg^−1^ single-layer graphene and 50 g kg^−1^ few-layer graphene; SL3 and FL3. 100 g kg^−1^ single-layer graphene and 100 g kg^−1^ few-layer graphene; SL4 and FL4. 150 g kg^−1^ single-layer graphene and 150 g kg^−1^ few-layer graphene; error bars indicate ± standard deviation. Different letters above error bars indicate significant difference between treatment (*p* < 0.05, LSD test).

### Soil available nutrients, and soil enzyme activity

**Figure 2:**
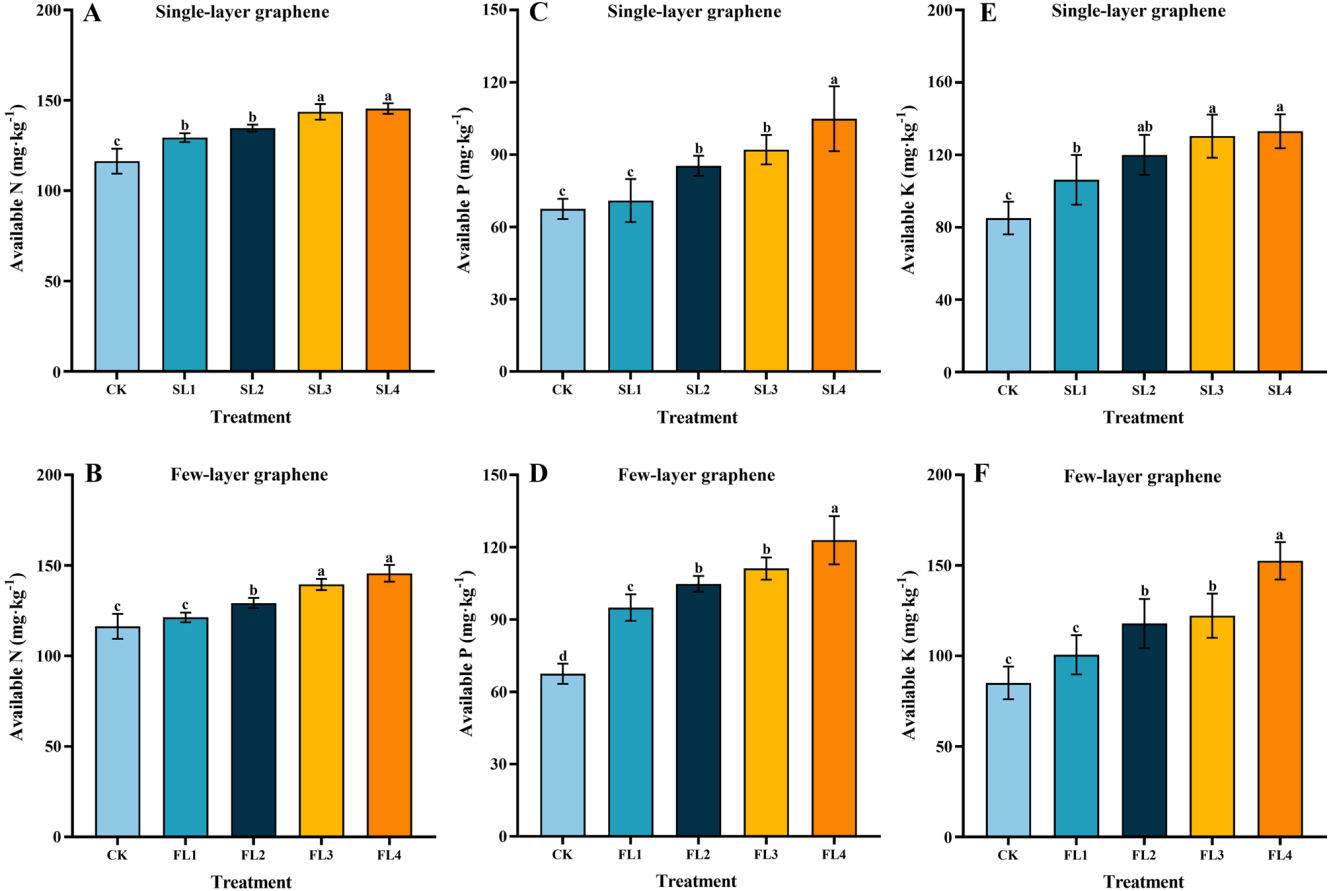
Effect of different graphene concentration and type on soil AN, AP, AK content. CK. 0 g kg^−1^ single-layer graphene and 0 g kg^−1^ few-layer graphene; SL1 and FL1. 25 g kg^−1^ single-layer graphene and 25 g kg^−1^ few-layer graphene; SL2 and FL2. 50 g kg^−1^ single-layer graphene and 50 g kg^−1^ few-layer graphene; SL3 and FL3. 100 g kg^−1^ single-layer graphene and 100 g kg^−1^ few-layer graphene; SL4 and FL4. 150 g kg^−1^ single-layer graphene and 150 g kg^−1^ few-layer graphene; (**A**,**C**,**E**) single-layer treatment; (**B**,**D**,**F**) few-layer treatment; error bars indicate ± standard deviation. Different letters above error bars indicate significant difference between treatment (*p* < 0.05, LSD test).

A few-layer graphene treatment had higher AN, AP, and AK contents than each corresponding treatment of single-layer graphene, indicating that graphene has the effect of limiting the release of available nutrients (Fig. 2). Soil AN, AP, and AK contents increased gradually as graphene application increased (Fig. 2A–F), and all of them were higher than the non-graphene control. Among these, the single-layer graphene SL4 treatment considerably raised the AN, AP, and AK contents compared to CK by 25.03%, 55.46%, and 56.34%, respectively. The AN, AP, and AK contents were dramatically enhanced by 25.19%, 82.17%, and 79.27%, respectively, compared to CK after the FL4 treatment with a few layers of graphene.

**Figure 3:**
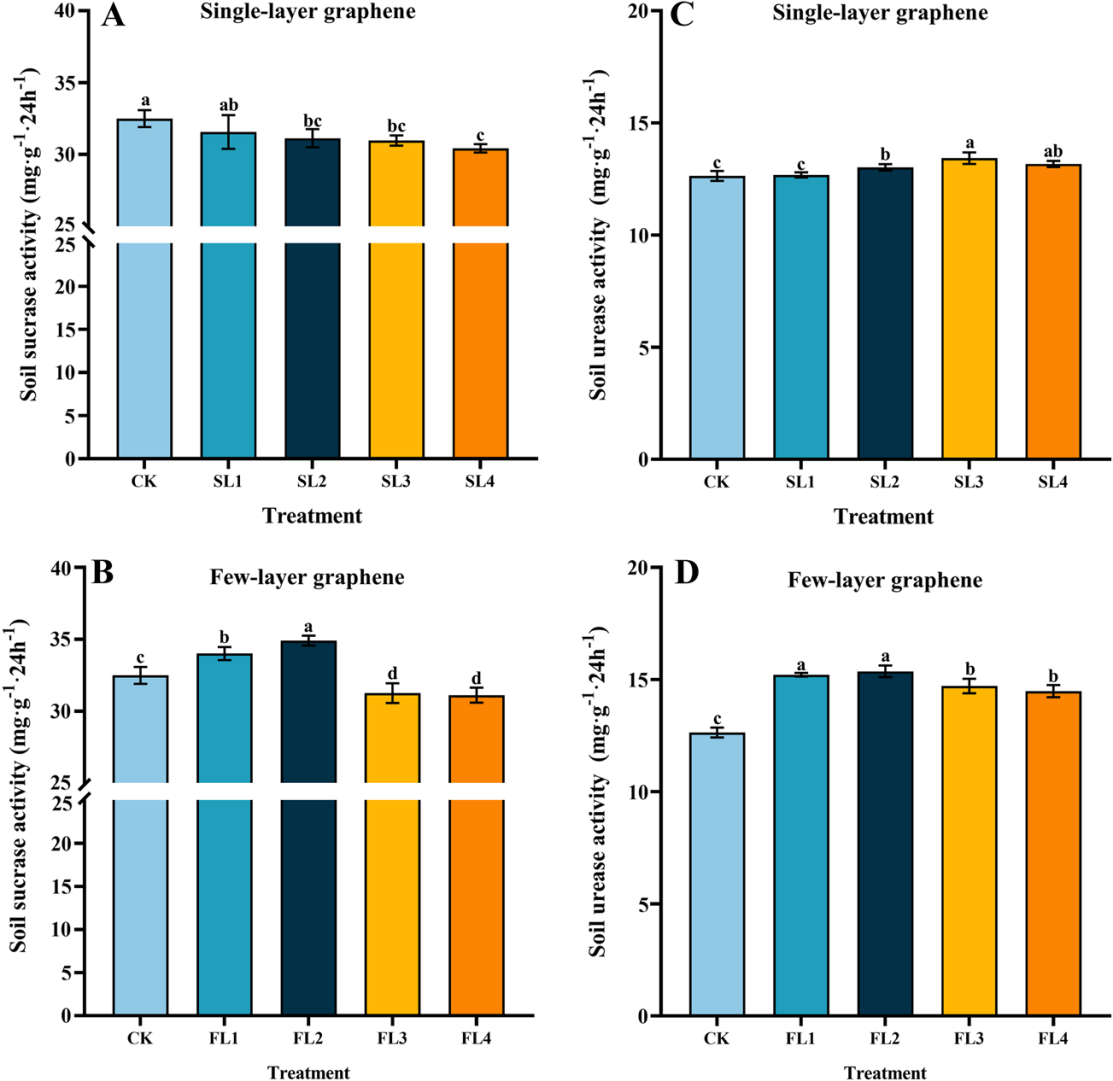
Effect of different graphene concentration and type on soil sucrase activity, urease activity. CK. 0 g kg^−1^ single-layer graphene and 0 g kg^−1^ few-layer graphene; SL1 and FL1. 25 g kg^−1^ single-layer graphene and 25 g kg^−1^ few-layer graphene; SL2 and FL2. 50 g kg^−1^ single-layer graphene and 50 g kg^−1^ few-layer graphene; SL3 and FL3. 100 g kg^−1^ single-layer graphene and 100 g kg^−1^ few-layer graphene; SL4 and FL4. 150 g kg^−1^ single-layer graphene and 150 g kg^−1^ few-layer graphene; (**A**,**C**) single-layer treatment; (**B**,**D**) few-layer treatment; error bars indicate ± standard deviation. Different letters above error bars indicate significant difference between treatment (*p* < 0.05, LSD test).

Figure 3A illustrates how single-layer graphene treatments decreased soil sucrase activity and demonstrated a decreasing trend with increasing concentration. SL2, SL3, and SL4 Treatment significantly decreased soil sucrase activity from 4.39%, 4.93%, and 6.80% compared to CK. Comparatively, increased sucrase activity was promoted by low concentrations of few-layer graphene (FL1 and FL2 treatments) (Fig. 3B), with significant increases of 4.66% and 7.43%, respectively, compared to CK. The FL3 and FL4 treatments considerably reduced the low sucrase activity in soil compared to CK by 3.96–4.45% as the concentration of few-layer graphene increased. When compared to single-layer graphene, it can be demonstrated that few-layer graphene promotes the accumulation of sucrase content better (Fig. 3A,B). As shown in Fig. 3C,D, the few-layer graphene treatment increased urease activity more than the single-layer graphene treatment. Graphene now improved soil urease activity, which first increased and subsequently reduced with increasing graphene application. With a considerable rise of 21.60% above CK, the FL2 therapy exhibited the greatest enzyme activity of all of them.

### TN, TP, and TK content of the plant

**Figure 4:**
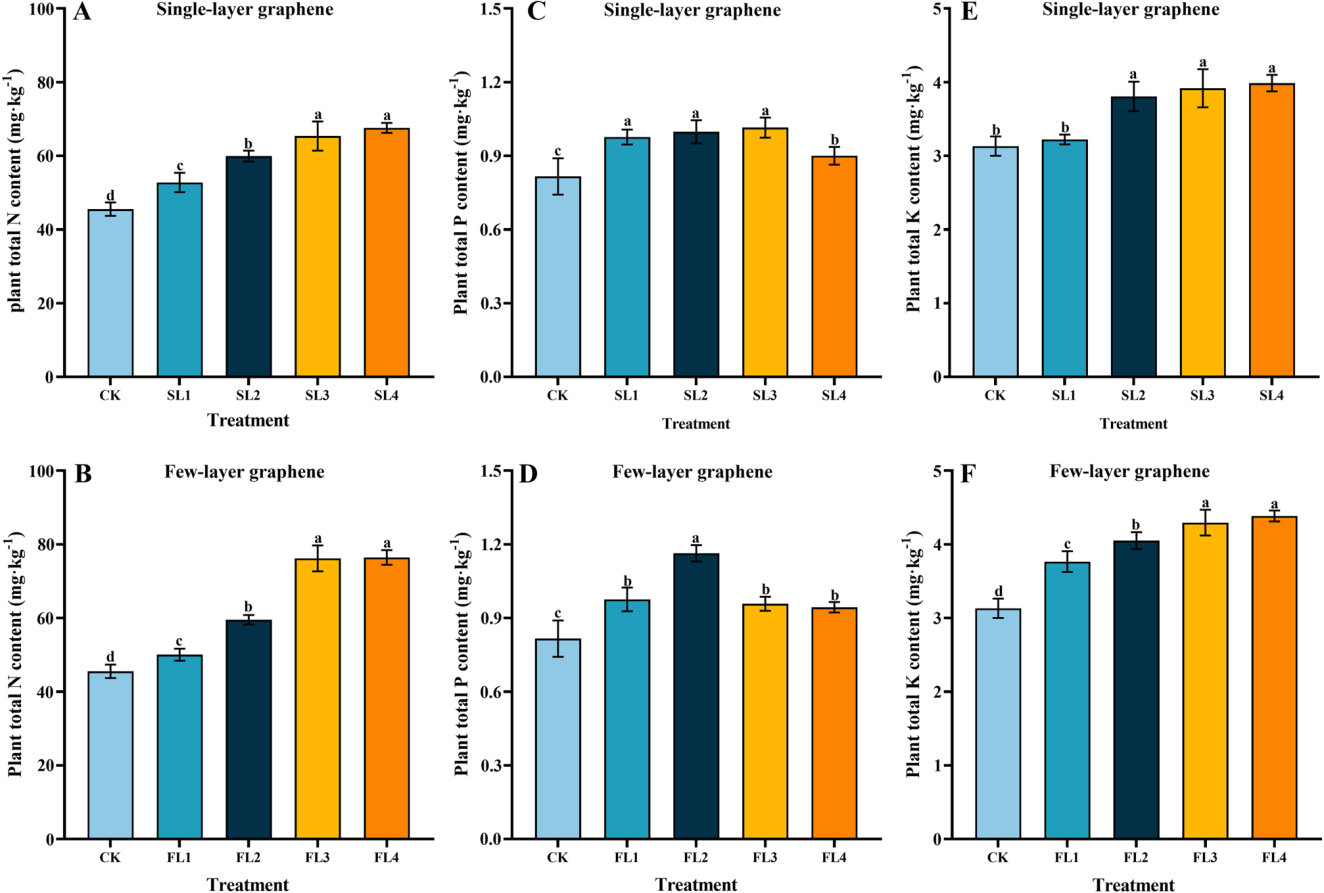
Effect of different graphene concentration and type on plant total N, total P and total K content. CK. 0 g kg^−1^ single-layer graphene and 0 g kg^−1^ few-layer graphene; SL1 and FL1. 25 g kg^−1^ single-layer graphene and 25 g kg^−1^ few-layer graphene; SL2 and FL2. 50 g kg^−1^ single-layer graphene and 50 g kg^−1^ few-layer graphene; SL3 and FL3. 100 g kg^−1^ single-layer graphene and 100 g kg^−1^ few-layer graphene; SL4 and FL4. 150 g kg^−1^ single-layer graphene and 150 g kg^−1^ few-layer graphene; (**A**,**C**,**E**) single-layer treatment; (**B**,**D**,**F**) few-layer treatment; error bars indicate ± standard deviation. Different letters above error bars indicate significant difference between treatment (*p* < 0.05, LSD test).

According to Fig. 4, the overall trend of plant nutrients rose as graphene application increased. Among them, the concentrations of TN and TK contents displayed a similar trend, both of which rose with increasing concentration, and each treatment reached a significant difference level from CK (Fig. 4A,B,E,F). Accordingly, TP content peaked in the SL3 and FL2 treatments before declining with the increasing application, albeit it remained considerably greater than the control. Overall, compared to the single-layer graphene treatment, the promoting effect of each treatment of few-layer graphene on the increase of plant nutrient content was better.

### Plant height, stem diameter, fresh weight, dry weight, and plant nutrients accumulation

According to Table 3, treatments SL1 and SL2 significantly increased the above-ground fresh and dry weight of maize plants compared to control (CK), and with increasing concentrations, treatments SL3 and SL4 slightly decreased the fresh and dry weight compared to control (CK) but did not reach a significant difference level. There were no appreciable variations across treatments in the effects of varying concentrations of single-layer graphene added to soil on plant height and stem diameter. The plant nutrient absorption was in line with the aforementioned findings (Table 3), peaking at the SL2 treatment with increases in N, P, and K uptake of 80.01%, 69.39%, and 66.67%, respectively, above CK.

**Table 3:** Effect of different graphene concentration and type on plant morphology and dry matter content.

Graphene	Treatment	Fresh weight (g)	Dry weight (g)	Plant height (cm)	Stem diameter (mm)
Single-layer graphene	CK	4.845 ± 0.460c	0.603 ± 0.022b	41.125 ± 1.560a	5.201 ± 0.054b
	SL1	5.465 ± 0.162b	0.735 ± 0.091a	43.425 ± 4.914a	5.730 ± 0.482ab
	SL2	7.350 ± 0.391a	0.825 ± 0.072a	44.350 ± 1.592a	6.435 ± 0.272a
	SL3	4.520 ± 0.162c	0.593 ± 0.067b	39.725 ± 1.161a	5.985 ± 0.104ab
	SL4	4.458 ± 0.139c	0.588 ± 0.026b	39.400 ± 0.442a	5.994 ± 0.058ab
Few-layer graphene	CK	4.845 ± 0.460c	0.603 ± 0.022c	41.125 ± 1.560b	5.202 ± 0.054a
	FL1	5.493 ± 0.157bc	0.775 ± 0.068bc	45.200 ± 1.429ab	6.053 ± 0.156a
	FL2	5.683 ± 0.142b	0.895 ± 0.106ab	46.825 ± 1.590a	5.660 ± 0.335a
	FL3	5.493 ± 0.157bc	0.775 ± 0.068bc	45.200 ± 1.429ab	6.053 ± 0.156a
	FL4	5.620 ± 0.099b	0.830 ± 0.023b	46.250 ± 1.195a	5.918 ± 0.127a

CK. 0 g kg^−1^ single-layer graphene and 0 g kg^−1^ few-layer graphene; SL1 and FL1. 25 g kg^−1^ single-layer graphene and 25 g kg^−1^ few-layer graphene; SL2 and FL2. 50 g kg^−1^ single-layer graphene and 50 g kg^−1^ few-layer graphene; SL3 and FL3. 100 g kg^−1^ single-layer graphene and 100 g kg^−1^ few-layer graphene; SL4 and FL4. 150 g kg^−1^ single-layer graphene and 150 g kg^−1^ few-layer graphene; error bars indicate ± standard deviation. Different letters above error bars indicate significant difference between treatment (*p* < 0.05, LSD test).

The growth of maize plants was accelerated by various concentrations of few-layer graphene; plant fresh weight, dry weight, plant height, and stem diameter all increased and then decreased with the concentration of graphene, and all of them were higher than the control, peaking at the FL2 treatment (Table 3). The plant's absorption of nutrients matched the trend of the plant growth indices (Table 4), and generally, the high concentration of the few-layer graphene treatment had a larger growth-promoting impact than the single-layer graphene treatment.

**Table 4:** Effect of different graphene concentration and type on plant nutrients accumulation.

Graphene	Treatment	N accumulation (g plant^−1^)	P accumulation (g plant^−1^)	K accumulation (g plant^−1^)
Single-layer graphene	CK	27.43 ± 2.10c	0.49 ± 0.03d	1.89 ± 0.19c
	SL1	38.79 ± 5.35b	0.72 ± 0.08b	2.37 ± 0.33b
	SL2	49.40 ± 3.71a	0.83 ± 0.11a	3.15 ± 0.41a
	SL3	38.66 ± 4.18b	0.60 ± 0.07c	2.31 ± 0.21b
	SL4	39.69 ± 1.60b	0.53 ± 0.02cd	2.34 ± 0.15b
Few-layer graphene	CK	27.43 ± 2.10d	0.49 ± 0.03c	1.89 ± 0.19c
	FL1	45.02 ± 11.91c	0.88 ± 0.22ab	3.36 ± 0.78b
	FL2	46.06 ± 7.74c	0.90 ± 0.17ab	3.14 ± 0.56b
	FL3	80.54 ± 8.67a	1.01 ± 0.13a	4.55 ± 0.62a
	FL4	63.47 ± 4.19b	0.78 ± 0.06b	3.64 ± 0.23b

CK. 0 g kg^−1^ single-layer graphene and 0 g kg^−1^ few-layer graphene; SL1 and FL1. 25 g kg^−1^ single-layer graphene and 25 g kg^−1^ few-layer graphene; SL2 and FL2. 50 g kg^−1^ single-layer graphene and 50 g kg^−1^ few-layer graphene; SL3 and FL3. 100 g kg^−1^ single-layer graphene and 100 g kg^−1^ few-layer graphene; SL4 and FL4. 150 g kg^−1^ single-layer graphene and 150 g kg^−1^ few-layer graphene; error bars indicate ± standard deviation. Different letters above error bars indicate significant difference between treatment (*p* < 0.05, LSD test).

### Correlation analysis

**Figure 5:**
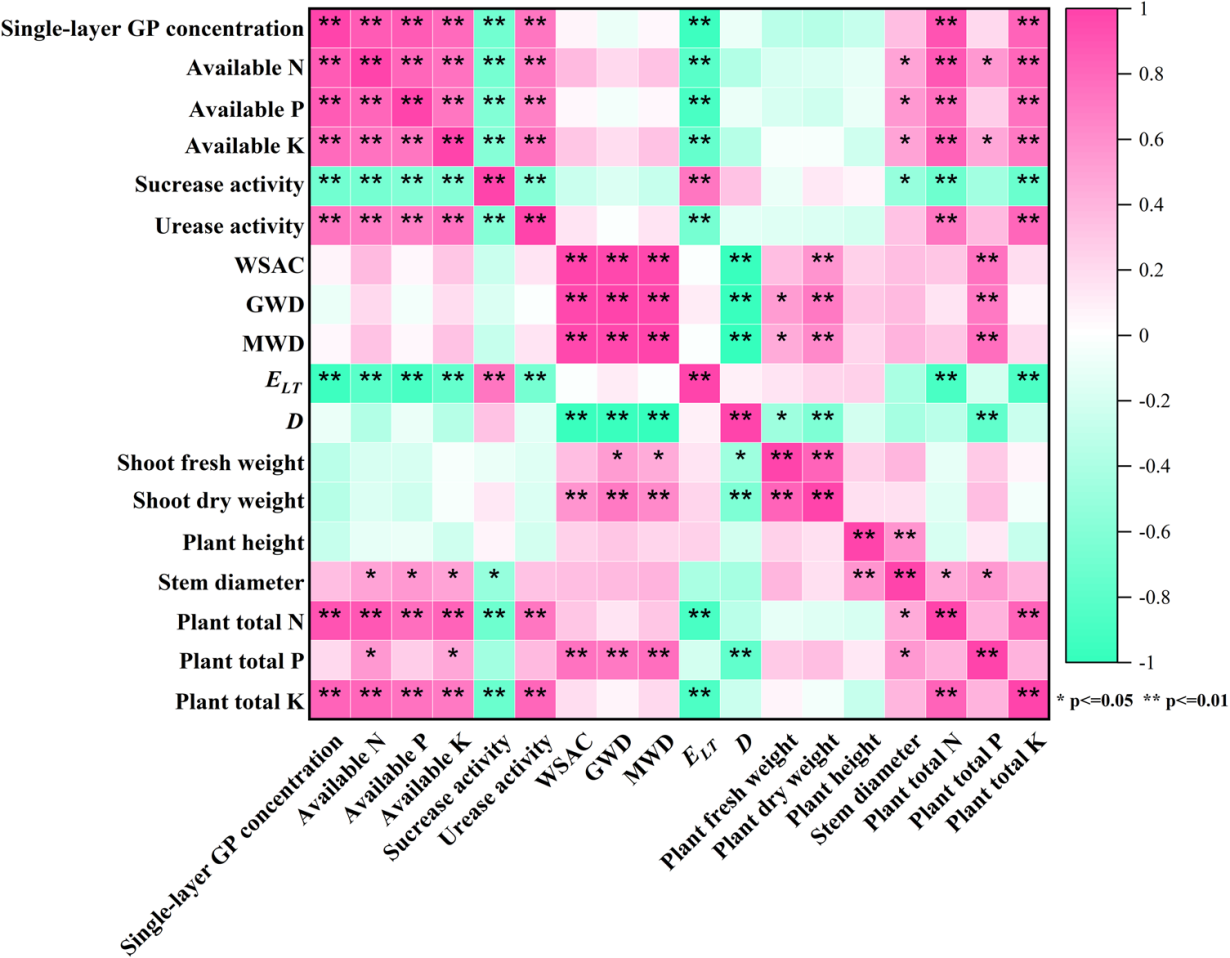
Correlation analysis among the indicators of single-layer graphene treatments.

Figure 5 shows that single-layer graphene was significantly positively correlated with soil available nutrients content and urease activity, but not with soil aggregates WSA, GMD, and MWD. WSA, GMD, and MWD were found to be significantly unrelated to *E*_*LT*_ and insignificantly related to *D*. The use of graphene-enhanced soil aggregate structure, activated soil nutrients, increased soil enzyme activity, and was directly responsible for the increase in soil accessible nutrients content. There was no significant correlation between single-layer graphene and plant growth, but there was a significant positive correlation between total plant nitrogen and total plant potassium, as well as a positive correlation between soil-accessible nutrient content and plant N and K, indicating that the use of single-layer graphene increased soil available nutrients, which promoted plant growth.

**Figure 6:**
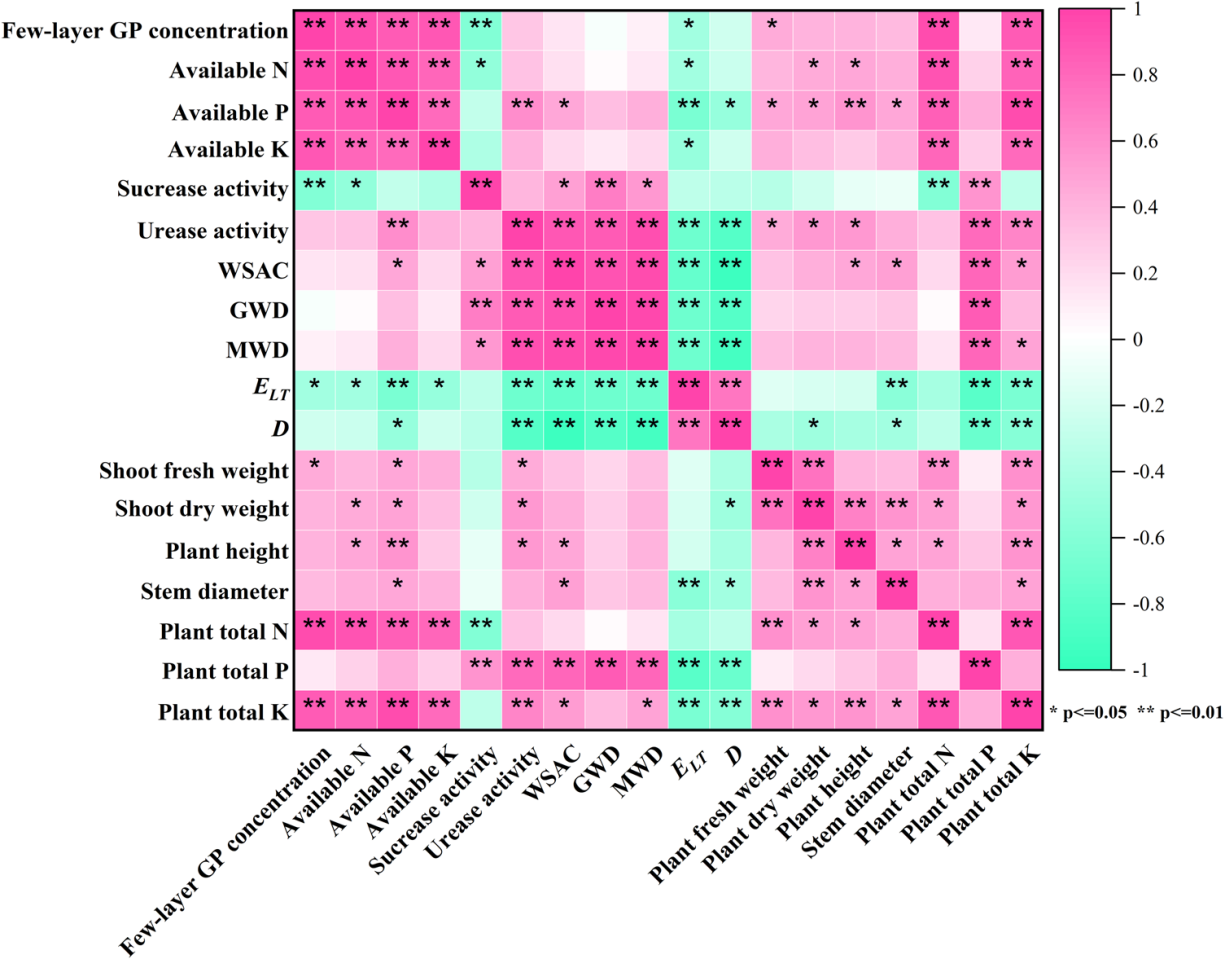
Correlation analysis among the indicators of few-layer graphene treatments.

As illustrated in Fig. 6, few-layer graphene and soil nutrients were significantly positively correlated with WSA, GMD, and MWD, but not with WSA, GMD, MWD, and *E*_*LT*_, and not with *D*. TN and TK were considerably and positively connected with few-layer of graphene and soil nutrients, but not with plant height, stem diameter, or dry matter accumulation; AN and AP were significantly correlated with plant height, stem diameter, and dry matter accumulation. It appears that graphene boosts nutrient absorption by plants by increasing the available nutrient content of the soil, which ultimately promotes plant development.

## Discussion

Soil agglomerates are clumps of soil particles cemented into granules and small clumps, and largely into spheres, whose number and distribution are key factors affecting the stability of soil structure, and the distribution of each particle level can be used to characterize the erosion resistance of soil^30^. The GMD, the MWD, and the mass fraction of soil big agglomerates (> 0.25 mm) give a comprehensive picture of soil agglomeration^36,37^. Water-stable agglomerates with a diameter of > 0.25 mm are thought to be helpful for water sequestration, nutrient absorption and release, and root respiration^38^. Adding carbon-based materials to soils has been demonstrated to alter the morphology of soil agglomerates and stimulate the creation of large-size agglomerates, which improves soil quality and increases the stability of soil agglomeration structure^39,40^. The findings showed that, when compared to the control (CK), adding single-layer graphene and few-layer graphene to the soil promoted the stabilization of soil large agglomerates while increasing their content in the soil and inhibiting agglomerate disruption, both at 25 g kg^−1^ (SL2 and FL2) and 50 g kg^−1^ (SL3 and FL3), and few-layer graphene increased soil large agglomerates. It shows that incorporating different layers of graphene into the soil can cause dispersed soil particles to clump together to form water-stable agglomerate particles that are beneficial to crop growth and development, improve agglomerate stability, and contribute to the improvement of soil structure. For > 0.25 mm agglomeration formation, the single-layer graphene treatment outperforms the few-layer graphene treatment.

It was noted that higher MWD and GMD values indicate higher soil agglomerate stability. At the same time, the better and more stable the agglomerate structure is, the smaller the *D* value. On the contrary, the higher the value, the soil texture is more viscous and thick, the soil is less porous^37^. Single-layer graphene and few-layer graphene increased the MWD and GMD of soil water-stable agglomerates in this study, their values were always higher than CK with increasing graphene concentration, indicating higher mean particle size agglomeration and greater agglomerate stability with larger values of MWD and GMD. This is in line with Yang and Lu^30^. in their study of carbon-based materials on soil agglomerates, which could be because, on the one hand, carbon-based materials stimulate microbial activity when applied to soil, resulting in secretions that promote the agglomerate formation, forming more agglomerate cementing substances, and increasing agglomerate stability^41^. On the other hand, graphene's highly developed pore structure and enormous specific surface area enable it to adsorb and immobilize inorganic ions and organic molecules in soil, generating organic–inorganic complexes and large particle-size agglomerates in the process. We thus postulate that graphene helps to enhance soil structure, increase the stability of agglomerates, and activate soil enzyme activity, which in turn helps to improve the ability to sequester available nutrients in the soil, therefore supplying nutrients to plants. The correlation analysis showed that the effect of few-layer graphene on soil aggregates was better than that of single-layer graphene treatment. Few-layer graphene showed an insignificant correlation with WSA, GMD and MWD and a significant negative correlation with *E*_*LT*_. WSA, GMD, and MWD showed a highly significant negative correlation with *E*_*LT*_ and *D*. The above results indicate that the effect of few-layer graphene application is more likely to promote the gelling and aggregation of soil agglomerates and improve their structural stability compared with single-layer graphene treatment. At the same time, few-layer graphene can also create good conditions for the agglomeration and aggregation of small and medium-sized soil particles, which can improve the structural stability and erosion resistance of soil through the adhesion and aggregation of soil particles with their own larger pores, thus providing a good growing environment for plants.

Because of their huge surface area and ability to absorb a large number of chemical ions and water molecules, carbon-based compounds improve the soil's fertilizer retention capacity. The addition of graphene nanoparticles to fertilizers can improve soil mucilage content, soil texture, soil nutrient absorption, soil electrochemical characteristics, and root nutrient absorption, consequently enhancing fertilizer utilization and minimizing chemical pollution from agricultural fertilizers. It can also improve the electrochemical characteristics of soil and boost nutrient uptake by roots, resulting in better fertilizer utilization, reduced chemical pollution from agricultural fertilizers, and increased efficiency^42,43^. One of the most important components of plant growth is nitrogen, which is mostly found in the form of alkaline nitrogen in the soil (NH_4_^+^-N and nitrate nitrogen)^44,45^. Because nitrate nitrogen is negatively charged and cannot be absorbed by soil colloids, it is easily lost with water, but NH_4_^+^-N in an AN solution is quickly lost due to volatilization^46^. It was found that the application of nanocarbon to the soil reduced the nitrate nitrogen leaching due to its water uptake and holding effect^47^. At the same time, because of the structural properties of nanocarbon, it has an adsorption effect on soil NH_4_^+^-N, which reduces NH_4_^+^-N volatilization^48,49^. In this experiment, the soil’s alkaline nitrogen content was positively correlated with the amount of graphene after single-layer graphene and few-layer graphene fertilizer application, indicating that both single-layer graphene and few-layer graphene had the effect of enhancing soil AN uptake and effectively supplementing the nitrogen nutrients required for plant growth. Simultaneously, graphene can increase the absorption and retention impact of phosphorus fertilizer in the soil, reducing nutrient loss and improving soil fertility in this experiment. This might be because graphene transforms the shape and activates the phosphorus activity in the soil via adsorbed metal cations, increasing the fraction of AP in the soil. It can enhance soil structure, and contribute to water and soil conservation by slowing the release rate of nutrients in the soil. Potassium, one of the necessary elements for crop growth, not only improves crop quality but also increases plant resilience to pests and diseases^50^. AK is the most common type of potassium taken up by plants in the soil, however, AK is weakly adsorbed and extremely mobile, resulting in limited potassium usage by plants^51,52^. Nanomaterials may react with potassium in a complicated way due to their huge specific surface area and strong adsorption characteristics^53^, significantly enhancing potassium uptake by plants. In this study, graphene increased the AK content in the soil, which was significantly higher than the control, demonstrating the K potentiation effect of graphene on the soil.

Nanocarbon material is an inorganic substance that can regulate the soil microenvironment and has a certain activating effect on soil enzyme activity, and it has been reported that it can regulate the soil microenvironment and a certain activating effect on soil enzyme activity^54,55^. Soil urease is a hydrolytic enzyme that catalyzes the hydrolysis of urea to create ammonia, carbon dioxide, and water. It is primarily engaged in the conversion of soil nutrients. Soil urease is a hydrolytic enzyme that catalyzes the hydrolysis of urea to create ammonia, carbon dioxide, and water. It is primarily engaged in the conversion of soil nutrients^56,57^. The results of this experiment showed that single-layer graphene and few-layer graphene could effectively enhance soil urease activity, proving that the soil AN content increased significantly after the addition of graphene to the soil in this experiment (Fig. 2A,B). The increasing effect of few-layer graphene was greater than that of single-layer graphene, which could be because few-layer graphene is composed in the form of stacks. Meanwhile, urease activity reduced to varying degrees as the number of layers of graphene increased, although it was always higher than the control (CK). This may be because, on the one hand, the toxic effects of carbon nanomaterials at a certain dose may be due to the generation of oxidative stress or free radicals, causing lipid peroxidation damage and destruction of membrane structure, loss of normal cellular functions, and cell death or apoptosis, and on the other hand, the toxic effects of carbon nanomaterials at a certain dose may be due to the generation of oxidative^58^. Root secretion capacity and microorganisms both suffer as a result of graphene. Even though the soil is high in nitrogen, it is unable to break down and mineralizes, resulting in a reduction in urease activity. The decrease in urease activity, on the other hand, may be influenced by N, and P, with an increase in soil phosphorus nutrients being detrimental to soil urease activity, and all of the above could be the reasons for the decrease in urease activity in this study due to high graphene concentration.

Soil sucrase is closely related to soil nutrient content and can be used by soil organisms through the hydrolysis of sucrose and provide energy, which can reflect the utilization of soluble substances in soil and the accumulation and transformation of soil nutrients^59,60^. Organic matter boosted nanomaterial dispersion and increased adsorption sites on the surface of carbon nanomaterials, which led to the discovery that soil nutrients might promote nanomaterial adsorption to soil enzymes^55,61^. A similar outcome was achieved in this investigation, which found that at concentrations of 25 g kg^−1^ and 50 g kg^−1^, few-layer graphene boosted soil sucrase activity (FL1 and FL2 treatments). In the single-layer graphene treatment, on the other hand, the enzyme activity decreased with the increased application. This might be owing to the significant adsorption of both soil nutrients and soil enzymes onto carbon nanomaterials, which creates site rivalry between the two and hence reduces soil enzyme activity adsorption.

Plants can benefit from nanomaterials that help them absorb nutrients like N, P, and K^62^. Furthermore, the carbon nanoparticles are negatively charged and will absorb cations when put around plant roots, allowing them to adsorb and transport some of the cations required by plants^63^. The findings of this study revealed that when single-layer graphene and few-layer graphene were applied to the soil, both boosted total N, P, and K content in the plants, with few-layer graphene having a slightly higher degree of nutrient absorption than single-layer graphene treatment. Nutrient intake is the foundation of dry matter creation and accumulation, and an increase in nutrient uptake rate is dependent on an increase in dry matter accumulation rate. The addition of single-layer graphene and few-layer graphene to the soil boosted plant fresh weight and encouraged plant dry matter mass accumulation, with the impact of few-layer graphene on dry matter mass accumulation being higher than that of single-layer graphene treatment. This corresponds to the plant’s ability to absorb nutrients in this study, and the few-layer graphene increased the rate of N, P, and K accumulation in the plants as well as the amount of N, P, and K accumulation, demonstrating the critical role of nutrient accumulation in plant dry matter accumulation. When graphene is introduced to soil, it has a large specific surface area and a great adsorption capacity, and part of the graphene enhances the nutrients in the soil through adsorption^64^. Another part of graphene is adsorbed on the root surface because graphene's ion transport ability is extremely strong, it can act as an ion transport channel, allowing enriched ions to be quickly transported to the graphene surface of the roots and then absorbed by the roots. As a result, the addition of graphene can increase ion transport efficiency, nutrient absorption rate, and plant growth rate. When the amount of graphene injected surpassed a particular threshold (> 50 g kg^−1^), the extra graphene covered the root surface and inhibited nutrient delivery, resulting in a drop in plant height. When the amount of graphene injected surpassed a particular threshold (> 50 g kg^−1^), the extra graphene covered the root surface and inhibited nutrient delivery, resulting in a drop in plant height. Single-layer graphene at added concentrations of 100 g kg^−1^ and 150 g kg^−1^ (SL3 and SL4) were significantly lower than the control (CK), but not to the level of significant difference. The few-layer graphene treatment, on the other hand, exhibited a declining trend at 150 g kg^−1^ (FL4) but was still much greater than the control (CK). Meanwhile, although the graphene wrapped in the root system carries a large number of nutrients, the available nutrients in the soil gradually increased with the gradual increase of graphene concentration, but plant uptake did not increase at the same time (Table 4). Therefore, we speculate that the excessive graphene and there may be a counteraction of competing with each other for the root uptake sites with nutrients, nutrients were unable to reach the above-ground portion in time, affecting plant development.

## Conclusion

The addition of appropriate amount graphene to the soil activated soil nutrients, increased the content of available nutrients, improved the structure of soil aggregates, which improved plant uptake of nutrients, improved carbon and nitrogen metabolism, and indirectly promoted plant growth and dry matter accumulation. The positive effect of 50 g kg^−1^ few-layer graphene added to the conventional fertilizer application on soil nutrient conversion and seedling growth during the seedling stage of maize was greater than that of single-layer graphene, according to a comprehensive analysis, and the addition of 50 g kg^−1^ few-layer graphene to the conventional fertilizer application was beneficial to the formation of strong maize seedlings in the albic soil area.

## Data Availability

All data generated or analyzed during this study are included in this published article.
